# A Survey on Virtualization of Wireless Sensor Networks

**DOI:** 10.3390/s120202175

**Published:** 2012-02-15

**Authors:** Md. Motaharul Islam, Mohammad Mehedi Hassan, Ga-Won Lee, Eui-Nam Huh

**Affiliations:** 1 Internet Computing and Network Security (ICNS) Laboratory, Department of Computer Engineering, College of Electronics and Information, Kyung Hee University, Yongin-si 446-701, Korea; E-Mails: motahar@khu.ac.kr (M.M.I.); gawon@khu.ac.kr (G.-W.L.); 2 Research Chair of Pervasive and Mobile Computing, College of Computer and Information Sciences, King Saud University, P.O. Box 51178, Riyadh 11543, Saudi Arabia; E-Mail: mmhassan@ksu.edu.sa

**Keywords:** VSN, sensor substrate, 6LoWPAN, IP-WSN, SInP, SVNSP, ALU

## Abstract

Wireless Sensor Networks (WSNs) are gaining tremendous importance thanks to their broad range of commercial applications such as in smart home automation, health-care and industrial automation. In these applications multi-vendor and heterogeneous sensor nodes are deployed. Due to strict administrative control over the specific WSN domains, communication barriers, conflicting goals and the economic interests of different WSN sensor node vendors, it is difficult to introduce a large scale federated WSN. By allowing heterogeneous sensor nodes in WSNs to coexist on a shared physical sensor substrate, virtualization in sensor network may provide flexibility, cost effective solutions, promote diversity, ensure security and increase manageability. This paper surveys the novel approach of using the large scale federated WSN resources in a sensor virtualization environment. Our focus in this paper is to introduce a few design goals, the challenges and opportunities of research in the field of sensor network virtualization as well as to illustrate a current status of research in this field. This paper also presents a wide array of state-of-the art projects related to sensor network virtualization.

## 1. Introduction

Recent advances in wireless communications and electronics have enabled the development of low-cost, low-power, multifunctional sensor nodes that are small in size and communicate untethered over short distances. A sensor network consists of a large number of sensor nodes that are densely deployed either inside the phenomenon of interest or very close to it [1–5]. Due to the rapid advancement of electronics, tiny sensor nodes are capable of supporting IP protocol stack. 6LoWPAN facilitates the IPv6 communication over low power and low cost sensor nodes [5–7].

In the past, applications of sensor networks were thought to be very specific. The communication protocols of sensor networks were also very simple and straightforward. Some researchers were even against the use of the internetworking concept in WSNs for different reasons such as the resource constraints for layered architecture, the problems of configuring large numbers of devices, the essence of sensor nodes’ distinct identity, *etc.*, but with the advent of the Internet of Things and federated IP-WSNs, this demand is going to be blurred. The huge numbers of IPv6 addresses, the necessity for end to end communication and advances in micro-electronics have changed the concepts of the research community. Now a tiny sensor node can hold a compatible TCP/IP protocol stack, so we can now think of using the concept of internetworking protocols in IP-WSNs. We can easily think of providing IPv6 addresses to individual sensor nodes since it provides around 6 × 10^23^ addresses per square meter of the Earth’s surface.

IP-enabled sensor nodes have opened the door for further research into advanced and distributed applications in sensor networks [5]. The limitations of resource constraints of a tiny sensor node could not stop the advancement of research in the field of sensor networks. Now a tiny sensor node can also be identified by an individual IP address [6,7]. Recently, network virtualization has created a resonance among the network based research community. The concept of sensor virtualization has also attracted a great deal of attention from industry and academia [8–10]. Virtualization on sensor networks (VSN) can be defined as the separation of the function for the traditional wireless sensor network (WSN) service provider into two parts: sensor infrastructure provider (SInP) that manages the physical sensor infrastructure, and sensor virtualization network service provider (SVNSP) that develops the VSN by aggregating resources from multiple SInPs and offer services to the application level users (ALU).

The WSN virtualization renaissance has been caused mainly from the realization that most of the sensor nodes in a WSN remain idle for most of the time. Sensor network virtualization is one of the best ways to utilize the physical sensor node. Virtualization of sensor networks can provide a platform upon which novel sensor network architectures can be built, experimented and evaluated [11–17]. In addition, virtualization in WSNs is expected to provide a clean separation of services and infrastructure and facilitate new ways of doing business by allowing the trading of sensor network resources among multiple service providers and application level users [18–20].

This type of virtual sensor environment can be ensured from the coexisting heterogeneous WSN architectures that are free from the limitations of existing multi-vendor sensor networks [21]. The importance of sensor virtualization is manifold in this age of worldwide economic recession. VSN can provide cost effective and green technology solutions to design smart houses and cities. In this paper we survey the virtualization of wireless sensor network, discuss the challenges and opportunities. Finally we justify the application of VSN in different area such as in the battlefield scenario, in monitoring rock slides and animal crossing within a mountainous terrain, in designing and monitoring smart houses, structural mentoring, healthcare, vehicle telematics, agricultural monitoring and industrial monitoring. In the above mentioned application areas, the concept of VSN can be used to make the system cost effective. Although the application of VSN in a few of the areas such as agricultural monitoring may not be cost effective and suitable at present, with the evolution of the agricultural revolution it will be deemed appropriate in the near future.

The main contributions of this paper are as follows:

- We have proposed a novel business model of virtualization of sensor networks.
- We have surveyed the virtualization sensor networks in WSNs.
- We have discussed the design goals of virtualization technology in wireless sensor networks.
- We have studied and summarized the contemporary sensor network virtualization projects.
- We have figured out the challenges and opportunities of virtualization in WSNs.
- Finally we have depicted future research scopes and few major application areas of VSN.

The remainder of the paper is organized as follows: Section 2 reviews the background related to virtual sensor network overlay sensor networks, VSN and its business models and the existing protocols to support VSN. In Section 3 we discuss VSN design, which consists of the design goals and challenges behind VSN. Section 4 describes related works and a few contemporary sensor network virtualization projects. Section 5 discusses a few opportunities provided by VSN. Section 6 depicts the typical application areas related to VSN. Section 7 focuses on some discussions that include available solutions, future research scope, open issues and finally Section 8 concludes the paper.

## 2. Background

Virtualization of Sensor Network (VSN) is a brand new research approach in the field of Wireless Sensor Network (WSN). Before proceeding further, we need to clarify few basic concepts and the difference between traditional WSN, conventional Virtual Sensor Network, Overlay Sensor Network and VSN. In brief, a traditional wireless sensor network consists of a large number of sensor nodes that are densely deployed either inside the phenomenon of interest or very close to it [1]. In this paper VSN means virtualization of WSN as defined in the introduction and in Section 2.3. The term VSN in this paper is synonymously used for the process of virtualization of sensor network and for the network that support virtualization.

### 2.1. Virtual Sensor Network

In traditional sensor network, all the nodes in the network perform more or less as equal partners to achieve the goal of deploying sensor nodes [1,2]. Virtual Sensor Network consists of collaborative wireless sensor network. It is formed by a subset of sensor nodes of a wireless sensor network, with the subset being dedicated to a certain task or an application at a given time [19,21]. In contrast, the subset of nodes belonging to the virtual sensor network collaborates to carry out a given application at a specific time. A virtual sensor network can be formed by providing logical connectivity among collaborative sensor nodes. Nodes can be grouped into different virtual sensor networks based on the phenomenon they track or the task they perform. The virtual sensor network protocol should provide the functionality for network formation, usage, adaptation, and maintenance of subset of sensors collaborating on a specific task. Even the nodes that do not sense the particular event could be part of it as long as they are allowing sensing nodes to communicate through them [22].

### 2.2. Overlay Sensor Network

In traditional approaches, an overlay network is a computer network which is built on the top of another network. Different types of distributed systems such as cloud computing, peer-to-peer networks, and client-server applications are overlay networks because their nodes run on top of the Internet. The Internet was built as an overlay upon the telephone network [23]. An overlay sensor network is a type of sensor network that creates a virtual topology on top of the physical topology of a wireless sensor network. Nodes in an overlay network are connected through virtual links which correspond to paths in the underlying network. Overlays are typically implemented in the application layer, though various implementations at lower layers of the network stack do exist. In [24] the authors have proposed a novel access architecture using a sensor network overlay. By embedding sensing, computation and transmission capabilities, sensor nodes can cooperate to serve as an effective monitoring and data gathering technology. In the scheme, the transmission of control messages is done at the sensor plane, in parallel with data transmission in the data plane.

### 2.3. Virtualization of Sensor Network and its Business Model

**Figure 1.**
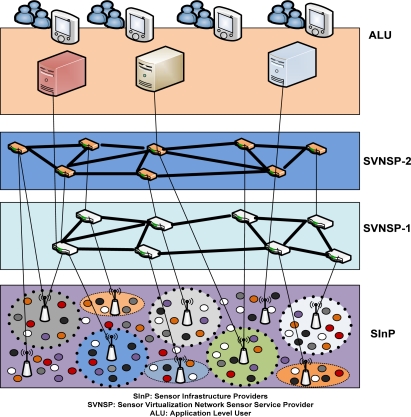
Business model of sensor network virtualization.

Unlike wireless sensor networks, the VSN environment has a collection of multiple heterogeneous sensor network resources that coexist in the same physical space. In Figure 1, there are different types of physical sensor networks existing in the same domain. There are many Sensor Infrastructure Providers (SInPs), indicated by different circles in the lower layer of Figure 1. There are two Sensor Virtualization Network Service Providers (SVNSPs) in the model. Each SVNSP hires resources from one or more SInPs to form VSNs, and deploys customized protocol and services.

In traditional wireless sensor networks the infrastructure provider and service provider are same entity, but increasingly diversified applications of sensor networks in different fields such as battlefield surveillance, habitat monitoring, disaster recovery and building smart homes, make it necessary to differentiate between the WSN infrastructure providers’ and service providers’ perspective. The objective behind this is to minimize the cost of establishment and to reduce the manageability cost. The main difference between the participants in the sensor network virtualization model and the traditional model is the presence of two different roles, SInP and SVNSP, as opposed to the WSN provider as a whole.

**SInP** It deploys and manages the substrate physical sensor network resources. They offer their resources through programmable interfaces to different SVNSPs. SInPs distinguish themselves through the type of services they provide and the sensor node of which vendor and communication protocol they used. Different Vendor Companies can deploy sensor nodes and make their individual infrastructure which can be used by the company or can be leased to different virtual service providing companies to run their individual applications. It helps the effective utilization of the physical sensor node on a broader scale.

**SVNSP** It leases resources from multiple SInPs to create and deploy VSNs by sharing allocated virtualized network resources to offer end to end application user services. A SVNSP can achieve network services from multiple InPs. The resources used by the SVNSP can be reused by the other SVNSPs in a recursive fashion.

**ALU** Application level users (ALUs) in the VSN model are similar to those of the existing WSNs, except that the existence of multiple SVNSPs from competing SInPs provides a wide range of choice. Any end user can connect to multiple SVNSPs from different SInPs for using multiple applications.

### 2.4. Existing Protocols to Support VSN

Currently there are different protocols that support VSN. The Melete system is based on the Mate virtual machine that enables reliable storage and execution of concurrent applications on a single sensor node [16,25]. Most recently the Federated Secure Sensor Network Laboratory (FRESnel) at the Cambridge University aims to build a large scale federated sensor network framework with multiple applications sharing the same sensor node resources [26]. The primary aim of this project is to offer an environment that can support multiple applications running on each sensor node [27]. It provides an execution environment that hides the system details from the running applications. The system operates in a shared environment. The key characteristics of this approach are: A virtualization layer that is running on each sensor node abstracts access to sensor resources and allows the management of these resources through policies expressed by the infrastructure owner. A runtime environment on each node allows multiple applications to run inside the node. A policy based application deployment that enables multiple applications to be deployed over the shared infrastructure.

## 3. Designing Virtualization of Wireless Sensor Network

### 3.1. Design Goals behind VSN

The design goals for successfully realizing virtualization in sensor network have been addressed by different research groups. In order to materialize the issues behind sensor network virtualization, each of these design criteria should be fulfilled.

#### 3.1.1. Flexibility

Flexibility means designs that can adapt when external changes occur. In designing VSNs we must pay heed to the flexibility issue. Virtualization in sensor networks must provide freedom for every aspect of sensor networking. Each sensor virtualization network service provider should be free to implement arbitrary virtual sensor network topologies. It should also provide flexibility in routing and forwarding functionalities, and customized control protocols that are independent of the underlying physical sensor network and other coexisting SVNSPs. For example, deploying source routing in today’s sensor network depends much on the consensus among the SInPs. In a virtualized environment, the owner of a SVNSP should be able to offer source routing without having to coordinate with any other parties. In short, source routing allows a sender of a packet to partially or completely specify the route; the packet takes through the sensor network.

#### 3.1.2. Network Heterogeneity

Heterogeneity is an important issue in designing VSNs. Heterogeneity in the context of sensor network virtualization comes mainly from two perspectives: first, heterogeneity of the underlying sensor networking technologies *i.e.*, sensor nodes of different vendors; second, each end-to-end SVNSP, created on top of that heterogeneous combination of underlying SInP, can also be heterogeneous. SVNSPs must be allowed to compose and run cross domain end-to-end VSNs without the need for any specific solutions. Underlying infrastructures must also be capable of supporting heterogeneous protocols and algorithms implemented by different SVNSPs. In addition, heterogeneity of end user devices must also be taken into account. In Figure 1, we have shown that different types of sensor node coexist in the same physical substrate sensor network. The heterogeneity is necessary in the designing federated sensor network since it provides multiple types of services. The heterogeneity can be manifested in terms of sensor node deployment or the sensor network that is formed by the heterogeneous sensor nodes.

#### 3.1.3. Isolation

Sensor Network virtualization must ensure isolation between coexisting VSNs to improve fault-tolerance, security, and privacy. Sensor network protocols are prone to misconfigurations and implementation errors. Sensor virtualization must ensure that misconfigurations in one VSN are contained within itself and do not affect other co-existing VSNs. Isolation allows logical separation of the VSNs although they coexist on same physical substrate sensor network. The device abstraction promotes strong software isolation: multiple instances of unmodified sensing applications use their virtual sensor as if it were a dedicated physical sensor. Strong isolation also extends performance: each user’s performance is a function of sharing the device’s resources. Sharing guarantees a minimum fraction of a sensor’s resources.

#### 3.1.4. Manageability

Managing VSN applications has always been a major part of VSN design. In VSNs each SVNSP remains independent over a federated physical sensor network. By separating SVNSPs from SInPs, sensor network virtualization will modularize network management tasks and introduce accountability at every layer of networking architecture [3]. It must provide complete, end to end control of the VSNs to the SVNSPs obviating the requirement of coordination across administrative boundaries as seen in the existing WSN domain.

#### 3.1.5. Scalability

Coexistence of multiple sensor networks is one of the fundamental principles of sensor network virtualization. Scalability comes as an indispensable part of this equation. SInPs must scale to support an increasing number of coexisting VSNs without affecting their performance. For designing a large scale federated sensor network, it is necessary to design the VSN scalable so that any type of modification or addition of further physical sensor network can be easily done.

#### 3.1.6. Stability and Convergence

Isolation ensures that faults in one VSN do not affect other coexisting VSNs, but errors and misconfigurations in the underlying physical network can also destabilize a sensor network virtualization environment. Moreover, instability in the SInPs can lead to instability of all the hosted VSNs. VSNs must ensure the stability of sensor virtualization environment and in case of any instability the affected VSNs must be able to successfully converge to their stable states.

#### 3.1.7. Programmability

To ensure flexibility and manageability, programmability of the virtual sensor network elements is an indispensable requirement. Only through programmability, SVNSPs can implement customized protocols and deploy diverse services. Two pressing questions in this respect must have satisfactory answers: how much programmability should be allowed? And how it should be exposed? A win-win situation must be found where programmability is easy, effective, and secure at the same time. For sharing the resources of the resource constrained sensor node programmability may provide an opportunity to do further research activities in VSNs.

#### 3.1.8. Legacy Support

Legacy support or backward compatibility has always been a matter of deep concern when deploying any new technology. Conceptually, sensor network virtualization can easily integrate legacy support by considering the existing WSN domain as just another VSN into its collection of sensor networks; but whether and how it can be done efficiently remains an open challenge. For this design goal the researchers should pay attention.

#### 3.1.9. Experimental and Deployment Facility

Before deployment, any geographically distributed federated sensor network service is typically designed and evaluated in test labs in a controlled environment. Since it is very expensive to mimic a production sensor network, tests are limited to simple topologies and traffic patterns that do not necessarily represent the real-world environment. Moreover, migration of a sensor network to a different condition can also be extremely painstaking. By developing the service in a separate virtual sensor network from the very beginning can electively alleviate these problems. In addition, deploying new end-to-end services could not be easier than deploying it on a separate virtual sensor network of its own.

### 3.2. Challenges behind Designing VSNs

In the recent past it was a general thinking that a tiny sensor node with its little processing capability could be applicable in very specific areas and very specific purposes, but with the rapid advancement of micro electro mechanical systems (MEMS), the perceptions of researchers have been dramatically changed. Nowadays IP-based WSNs and implementation of 6LoWPAN sensor nodes have been used in different fields such as health care, facility management, building and home automation, personal sports and entertainment, asset management, environmental monitoring, security and safety and industrial automation. In these entire applications the sensor network virtualization concept can be a challenging issue. To the best of our knowledge true virtualization concepts have not yet been introduced. A few research papers have been published, but these focused on the gateway-based virtualization among different sensor networks and the public or private internet, but the implementation of a true virtualization concept in a sensor network has many challenging issues, a few of which are discussed here.

#### 3.2.1. Interfacing

SVNSP uses physical resources from one or more infrastructure providers to create sensor virtual networks. SInP must provide well-defined interfaces to allow service providers to communicate and express their requirements. For interoperability, such interfaces should follow a standard that should be able to express sensor virtualization requests in terms of virtual sensor nodes and virtual links along with their corresponding attributes. Sensor web architecture can be a good example for monitoring interfaces.

#### 3.2.2. Impact on Sensor Devices Resources

Impact on sensor device resources when VSNs are supported is a great challenging issue. Sensor nodes are very much resource constraint devices. Most of the virtual machines for the VSNs are designed with this idea in mind. In VSN special types of sensor node such as imote2, gumstix with enough memory and processing power to support multiple applications. The operating system running on each sensing device is embedded Linux, a multitasking operating system.

#### 3.2.3. Managing Limited Resources

Resource allocation in a sensor network virtualization environment refers to static or dynamic allocation of virtual sensor nodes and links on physical nodes and paths, respectively. This is sometimes known as virtual sensor network embedding. Embedding of virtual sensor networks, with constraints on nodes and links, can be reduced to the NP-hard problem even when all virtual network requests are known in advance. The embedding problem has been discussed in many papers for the virtual networking environment, but the embedding approach of traditional networks is not compatible with WSNs for the lack of its storage, computing power and limited battery power. That’s why comprehensive research for efficient embedding of virtual sensor network requests to the physical WSN is needed. Although it may seem simple, it is really an important design goal to be considered for the virtualization of resource constrained sensor node.

#### 3.2.4. Resource Discovery

In order to allocate resources for requests from different virtual sensor service providers, infrastructure providers must be able to determine the topology of the sensor networks they manage as well as the status of the corresponding sensor network elements. Moreover, adjacent infrastructure providers must also share reachability information to be able to establish links between their networks to enable inter domain sensor virtual network instantiation.

#### 3.2.5. Virtual Sensor Nodes and Virtual Links

Commercial vendors have been promoting virtual edge routers and sensor gateways as tools for simplifying WSN domain design. The concept can be extended with programmability to create substrate edge routers that will allow each service provider to customize their virtual routers. Scalability of a sensor network virtualization environment is closely tied to the scalability of the physical edge routers. Commercial edge router vendors may implement routers that can hold multiple virtual sensor routers. To increase network manageability and to handle network failure, migration of virtual routers can be an effective solution, but finding probable destinations for a migrating virtual router is restricted by multiple physical constraints like change of latency, link capacity, platform compatibility issues, and even capabilities of destination physical routers. It remains an open research issue.

#### 3.2.6. Quality of Service and Quality of Experience

Quality of service and quality of experience are very important for all sorts of virtualizations. Quality of service is a measurement of network operating conditions such as noise or lost or dropped packets *etc.* Quality of Experience is a measurement used to determine how well that sensor network is satisfying the application level user requirements. To ensure these aspects in VSN are the big challenge.

#### 3.2.7. Security

VSN provides information from the physical sensor node to the application level users. In large scale federated wireless sensor networks information provided by the physical sensors is very important. Any sort of misuse may cause adverse effect on the application level users, so VSNs should provide security and authenticity mechanisms.

#### 3.2.8. Sensor Network Virtualization Economics

In traditional sensor network economics, the physical sensor node is the major commodity of interest, but in the sensor network virtualization counterpart, virtual sensor nodes are important as well. In such a marketplace, physical sensor infrastructure providers and sensors virtualization service providers maintain buyer–seller relationships with brokers acting as mediators between these two parties. Application level users also participate as buyers of services from different virtualization service providers. There are two general types of marketplaces: centralized and decentralized. Centralized marketplaces are efficient, but vulnerable against attacks and not scalable. On the other hand, fully decentralized marketplaces are extensible and fault-tolerant, but prone to malicious behavior and inefficiency. However, the work focuses mostly on sensor virtual links, leaving incorporating virtual sensor nodes to the economic model as an open challenge.

#### 3.2.9. Resource Scheduling

When establishing a virtual sensor network, a service provider requires specific guarantees for the virtual nodes’ attributes as well as the virtual links’ bandwidth allocated to its network. For virtual sensor routers, a service provider might request guarantees for a minimum packet processing rate of the CPU, specific storage requirements, and a lower bound on the size of the memory. On the other hand, virtual sensor link requests may range from best-effort service to fixed loss and delay characteristics found in dedicated physical links. To provide such guarantees and to create an illusion of an isolated and dedicated network to each service provider, infrastructure providers must employ appropriate scheduling algorithms in all of the sensor network elements. Existing system virtualization technologies provide efficient scheduling mechanisms for CPU, memory, storage, and network interface in each of the virtual machines running on the host sensor node. Sensor network virtualization can extend these mechanisms to implement resource scheduling in the physical infrastructure.

#### 3.2.10. Admission Control and Resource Usage Policy

Sensor infrastructure providers must ensure that resources are not over-provisioned to uphold QoS guarantees. Consequently, they have to perform accurate accounting and implement admission control algorithms to ensure that resources allocated to the virtual sensor networks do not exceed the physical capacity of the underlying physical sensor network. Existing solutions perform admission control while statically embedding virtual sensor networks. However, they do not allow dynamic resizing of allocated resources. In order to avoid constraint violations by distributed federated virtual sensor networks, distributed mechanisms must be employed to make sure that service providers cannot overflow the amount of resources allocated to them by direct or indirect means.

#### 3.2.11. Naming and Addressing

Due to potential heterogeneity of naming and addressing schemes in coexisting virtual sensor networks, end-to-end communication and universal connectivity is a major challenge in a sensor network virtualization environment. In addition, application level users can simultaneously connect to multiple virtual networks through multiple infrastructure providers using heterogeneous technologies to access different services. Incorporating support for such heterogeneity in multiple dimensions is a fundamental problem in the context of sensor network virtualization. A few recent research projects separate identities of end hosts from their physical and logical locations to add an additional level of new direction and with the help of a global identifier space, provide universal connectivity without revoking the autonomy of concerned physical and virtual sensor networks. However, while conceptually possible, this is not physically implementable due to excessive memory requirements. Therefore, one of the key research directions in naming and addressing is to find a viable global connectivity enabling a federated sensor framework.

#### 3.2.12. Dynamism and Mobility Management

The sensor network virtualization environment is highly dynamic. At a macro level, virtual sensor networks with shared interests can be dynamically aggregated together to create federations of virtual sensor networks. Multiple federations and virtual sensor networks can also come together to form virtual sensor network hierarchies. Aggregation and dissolution of control and data planes for macro level dynamism is an unresolved issue. At a micro level, mobility of end users from one physical location to another and migration of virtual sensor routers for operation and management purposes pose the biggest challenge. Finding the exact location of any resource or end user at a particular moment and routing packets accordingly is a complex research challenge that needs efficient solutions. In addition, sensor network virtualization allows end users to move logically from one virtual sensor network to another, which further complicates the problem.

#### 3.2.13. Virtual Sensor Network Operations and Management

Virtual sensor network operations and management has always been a great challenge for sensor network operators. Division of accountability and responsibilities among different participators in a sensor network virtualization environment promises increased manageability and reduced scopes for error. There are proactive and reactive mechanisms to enforce accountability for hosted virtual sensor networks. Considerable flexibility must be introduced from the level of sensor network operation centers to intelligent agents at sensor network elements, to enable individual service providers configuration, monitor, and control their virtual sensor networks irrespective of others. Since a virtual sensor network can span over multiple underlying physical sensor networks, applications must also be developed to aggregate information from diverse, often conflicting, management paradigms followed by participating sensor infrastructure providers. Introducing a common abstraction layer, to be followed by all the management software’s can be an effective solution.

Failures in the underlying physical sensor network components can give rise to cascading failures in the virtual sensor networks directly hosted on those components. For instance, a physical link failure will result in failures of all the virtual links that pass through it. Similarly, any physical sensor node failure might require re-installations of the entire service provider’s custom software’s. Detection and effective isolation of such failures as well as prevention and recuperation from them to stable states are all open research challenges.

## 4. Related Works and Contemporary VSN Projects

There have been various research works addressing sensor network virtualization but to the best of our knowledge there are no survey papers in the literature. In the past the research community mostly paid attention to different aspects of sensor networks, such as architecture, routing, energy efficiency, security, reliable transmission and data aggregation [1,2], but recently a good number of related research articles have been published in the field of virtualization of sensor networks [8,9,16,19,20,28–30]. Among the related researches most of them have two approaches. A few researchers have focused on gateway based VSN concepts. In VIP Bridge-based ubiquitous sensor networks [28,29] the authors have proposed an approach of using bridged to integrate several different sensor networks into one virtual sensor network. Gateway based sensor-grid applications are also discussed in [30–32]. Other researchers have focused on developing middleware based virtual machines. In [16] the authors proposed a tiny virtual machine for a Sensor Network called Maté. Its code is broken up into small capsules of 24 byte-long instructions allowing complex programs to be under 100 bytes. Maté is implemented on the top of TinyOS [33]. In [27] each application on a sensing device runs inside a sandbox environment where access to hardware resources is only available through the Virtualization Runtime. But in [16] the author proposed virtualization in sensor nodes that have focused on fully virtualizing the host operating system. Instead in [27], author allows the applications to run as native processes in a controlled environment.

In [25] authors proposed a system called Melete which is based on the Maté virtual machine. The Melete system enables reliable storage and execution of concurrent applications on a single sensor node [25,34]. Agilla [35] is based on Maté and extends the approach by providing mechanisms for better injection of mobile code into the sensor network to deploy user applications. Impala [36] is a middleware designed for the ZebraNet project [37] and its goal is to enable application modularity, and adaptability to dynamic environments.

In [21,38,39] the authors proposed a simple and robust virtual infrastructure for massively deployed wireless sensor networks that is simple and can be leveraged by a number of different protocols. In [17] the authors discussed dynamic resource discovery and programming of WSNs with logical neighborhoods in details. In [38] the concepts of sensor virtualization for heterogeneous sensor network platforms are proposed. Table 1 summarizes the research directions of sensor network virtualization in different contemporary projects.

### 4.1. Sensor Network Virtualization Projects

Recently network virtualization has been a popular topic among the networking research community. The flow of virtualization technology has also touched the field of wireless sensor networks. Although a lot of research papers focus on sensor network virtualization, very little work has been initiated till now that focuses on the true concept of sensor network virtualization. In this section we summarize the key characteristics of few project launched very recently. Some of the projects are directly related to true sensor virtualization and others focus on sensor virtualization technology as a whole.

#### 4.1.1. FRESnel

FRESnel [40] stands for Federated Secure Sensor Network Laboratory. FRESnel is focusing to build a large scale federated sensor network framework with different applications sharing the same resources. The importance of this project is to ensure reliable intra-application communication as well as a scalable and distributed management infrastructure. It also ensures orthogonality, privacy and application security. The evaluation of the proposal is primarily through a large scale federation of sensor networks over the Cambridge University campus. The sensor nodes monitor different aspects such as temperature, pollution, movement, *etc.* and the network will be running various applications belonging to different authorities in the city or the coverage area. The overall aim of the project is to provide a federated sensor network platform that could be used by multiple applications in a seamless and secure manner. To achieve the goal, the project has focus on the following technical points:

- It will develop a language for service level description and agreement for this kind of sensor network sharing. The language will be the basis of resource and application management and monitoring framework.
- It will develop methodology to enable dynamic resource allocation in a decentralized fashion. The techniques will be designed to take into account application needs and network resources. It will be adaptive to varying application demands, scalable in terms of network resources, and robust to resource failures.
- It will identify the mechanisms for dynamic partitioning of the sensor network into application-specific virtual sensor networks. These mechanisms should protect nodes belonging to different partitions against each other, especially during the transitive phase of dynamic repartitioning. Protection is in the form of privacy preservation and observation of resource allocation boundaries.
- It will develop distributed and scalable communication protocols that will allow the components of a single sensor application to communicate with one another in a reliable, secured and efficient way. The underlying collection of nodes, which belongs to the same virtual sensor network, will be able to configure itself dynamically to include new resources and heal from network failures.
- It will develop distributed algorithms for processing queries with very different quality-of-service requirements for accuracy and delay. In the presence of high query loads, these algorithms should gracefully degrade the quality of query answers giving priority to address the needs of critical applications. Privacy policies may also require that the specificity of certain query answers be limited.
- It will deploy a prototype for federated sensor network around the University of Cambridge campus, also partially integrating existing sensor networks. This will be a limited version of the envisioned CityNet application. It will span different colleges and will cover heterogeneous monitoring needs ranging from room usage and college security by using cameras), to air quality by using bike-mounted bio-sensors and traffic monitoring by using vehicle-generated GPS trajectories forwarded to fixed nodes installed in different colleges.

#### 4.1.2. Virtual Sensor Networks (VSNs)

This project is carrying out by Computer Network research laboratory by Colorado State University [41]. The project team has simulated a top-down clustering scheme, cluster tree based routing schemes, VSN self organization scheme on top of the cluster tree, and VSN based subsurface chemical plume monitoring system. Currently the project team members are working on random routing, virtual coordinates, and VSN support functions [41–43]. Virtual Sensor Networks are an emerging form of collaborative Wireless Sensor Networks (WSNs). It supports collaborative, resource efficient, and multi-purpose WSNs. These networks may involve dynamically varying subset of sensor nodes and/or users. A Virtual Sensor Network can be formed by providing logical connectivity among collaborative sensors. Nodes can be grouped into different VSNs based on the phenomenon they track (e.g., rock slides *vs.* animal crossing which is discussed in the Section 6.2) or the task they perform. Virtual Sensor Networks are expected to provide the protocol support for formation, usage, adaptation, and maintenance of subset of sensors collaborating on a specific task(s). It should make efficient use of intermediate nodes, networks, or other Virtual Sensor Networks to deliver messages across members of it. The main idea of Virtual Sensor Networks is collaboration and resource sharing. By doing so, nodes achieve application objectives in a more resource-efficient way.

#### 4.1.3. SensorPlanet

SensorPlanet [44] is Nokia-initiated cooperation. It facilitates a global research framework on mobile device-centric large-scale wireless sensor networks. The outcomes of SensorPlanet are: (1) It gives a test platform that enables the collection of sensor data on a never seen scale, (2) It also provide central repository for sharing the collected sensor data for research purposes. The idea behind the initiative is that the participating universities will develop their own applications for example environment and traffic monitoring, urban and participatory sensing, wellness and navigation applications, *etc.* It will also share the collected data in order to conduct research on data analysis and mining, visualization, machine learning, *etc.*

Moreover, it is the goal to develop novel application ideas and use cases based on the learning and the research. The objectives these research frameworks are: (i) strengthening mobile device-centric Wireless Sensor Network research, establishing a new field within WSN; (ii) establishing an open source community around Wireless Sensor Networks; (iii) providing a forum for publishing early results. (iv) accelerating the concept of innovation and finding of new application areas; (v) creating an ecosystem for industry and academia collaboration; (vi) enabling research groups to design and implement large scale experiments; (vii) facilitating creation and sharing of large data sets; (viii) studying future interaction modes for accessing sensor data; (ix) developing novel abstraction and visualization methods for user friendly interaction; (x) investigating the ways of fusion methods where different sources provide different type of data on various levels of granularity and semantics.

#### 4.1.4. ViSE

The span of work for this project [45] is to extend an outdoor, wide-area sensor/actuator network test bed to support slivering and utilize a GENI candidate control framework, and then bring it into an environment of GENI federated test beds. This includes: (1) virtualization of the sensor/actuator system; (2) integration with GENI-compliant software artifacts, including the use of shirako software as the base for the control framework; (3) making the test bed publicly available to GENI users, starting in first year, and integrate it into an environment of GENI federated test beds by the end of second year; (4) providing documentation for test bed users, administrators, and developers. The project already completed an initial research-grade implementation of sensor virtualization in Xen that applies the approach to Pan-Tilt-Zoom video cameras. Right now, the project is targeting two types of users for the test bed. The first type is users that wish to experiment with long distance 802.11b wireless communication. Long-distance links are difficult to setup because they require access to towers and other infrastructure to provide line-of-sight. The second type of user is radar researchers. It can leverage the radar deployment. They are working with students from University of Puerto Rico and other researchers in Collaborative Adaptive Sensing of the Atmosphere (CASA) research center of University of Massachusetts to interpret and improve the quality of the radar’s data and test them for detection algorithms. They are soliciting feedback from these users about what they need to do on these nodes, and how the test bed can satisfy their needs. It is worthy notification that the test bed interacts with a remote clearing house to facilitate resource allocation.

#### 4.1.5. DVM

The goal of the project [46] is to build a system that supports software configuration in embedded sensor networks which support multiple levels. The system architecture is based on a dynamic operating system for sensor network [47]. It is an operating system that contains different components such as: (i) a fixed tiny static kernel (ii) binary modules that can be dynamically inserted, updated or removed unobtrusively. On top of operating system, there is a dynamically extensible virtual machine that interprets high-level scripts. Any binary module that is dynamically inserted into the operating system can register custom extensions to the virtual machine. Therefore, the high-level scripts that are executed by the virtual machine can efficiently access services that are exported by a module and tune module parameters. Overall these systems permit the flexibility of selecting the most appropriate level of reconfiguration.

#### 4.1.6. PRESTO

**Figure 2.**
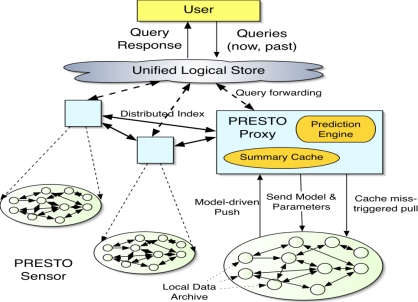
The PRESTO data management architecture.

PRESTO is a Predictive Storage Architecture for Sensor Networks [48,49]. Nowadays with the advent of sensor-cloud and sensor-grid architecture, we have seen a tremendous growth in extending the reaches of the Internet to numerous sensor data sources including RFIDs, weather and habitat monitors, building monitors, remote sensing data such as radar and others. These sensor data sources span a spectrum of power, data rate and platform requirements, from passive RFIDs and battery-powered wireless sensors to high bandwidth radar nodes. A unifying constraint across the spectrum of sensor data management applications is that the remote sensor nodes at the network edge are usually constrained in power, functionality and/or bandwidth, and communicate using multi-hop routing to a resource-rich proxy that connects the sensors to the Internet. PRESTO takes a fresh look at the design of tiered large-scale sensor networks that comprise tethered and untethered elements. It focuses three major questionnaires: (i) where should sensor data be archived? (ii) How can low-latency, interactive query processing be supported in-spite of the power and bandwidth constraints of sensors, frequent sensor failures and vagaries of wireless links? (iii) How can a user access data generated at numerous sensors and across a large geographic domain in an efficient manner? There are five main characteristics of PRESTO. It is discussed by the following features with the help of Figure 2.

- Tiered Design: PRESTO focus the idea that scalable sensor networks of the future will have multiple tiers, with several tens of untethered sensors per tethered sensor proxy and several tens of sensor proxies per application.
- Predictive Storage: It makes broad use of predictive techniques that easily fit to the correlated behavior of the physical world. It exploits technology trends in storage to build an architecture that emphasizes archival at remote sensors. It also uses extensive modeling and predictive caching at proxies.
- Interactive Usage: PRESTO data management architecture is designed for fast responses for *ad-hoc* queries on distributed sensor data without acquiring the energy costs of the data streaming approach or the losses, latency and reliability concerns to the remote sensors.
- Archival Queries: PRESTO supports archival queries on data resources which is very interesting. The ability for historical data query is important in many sensor applications to conduct unexpected and unusual events to better understand them for the future.
- Single Logical View of Data: PRESTO architecture provides a single logical view of data which is distributed across many sensor proxies and numerous remote sensors. This type of view can abstract the user from the variabilities at many levels lossy and unreliable remote sensor network. It also concerns spatial and temporal consistency issues in the sensor data as well as bandwidth and connectivity issues in the case of wireless proxies.

#### 4.1.7. SensEye

SensEye is a VSN related project that deals with multi-tier, multi-modal camera sensor network [50–52]. The speed of technological growth has led to the emergence of a variety of sensors, actuators, and networked sensor platforms. Nowadays networked sensors cover the spectrum of cost, form-factor, resolution, and functionality. For example, we can consider camera sensors, where available products range from expensive pan-tilt-zoom cameras to high-resolution digital cameras, and from inexpensive web-cams and cell-phone-class cameras to tiny cameras such as Cyclops. A similar set of options are becoming available for sensor platforms, with choices ranging from embedded PCs to PDA-class stargates, and from low-power motes to even lower power systems-on-a-chip. Multi-tier multi-modal networks provide an interesting balance of cost, coverage, functionality, and reliability. For instance, the lower tier of such a system can employ cheap, untethered elements that can provide dense coverage with low reliability. However, reliability concerns can be mitigated by seeding such a network with a few expensive, more reliable sensors at a higher tier to compensate for the variability in the lower tier. Similarly, a mix of low-fidelity, low-cost sensors and high-fidelity high-cost sensor can be used to achieve a balance between cost and functionality. Application performance can also be improved by exploiting alternate sensing modalities that may reduce energy requirements without sacrificing system reliability.

**Figure 3.**
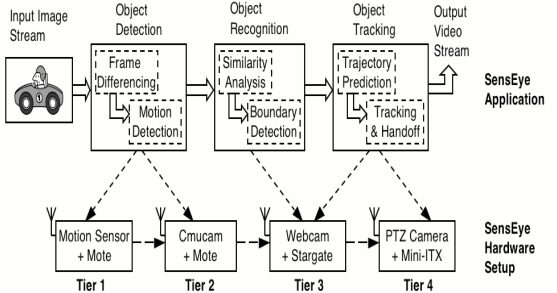
Software architecture of SensEye.

This project assumes a camera sensor network comprising multiple tiers which is depicted in Figure 3. A sensor node within each tier is assumed to be equipped with a camera sensor, a micro-controller, and a radio as well as on-board RAM and flash memory. Nodes are assumed to be battery-powered, and consequently, the overall constraint for each tier is energy. Within each tier, nodes are assumed to be homogeneous, while different tiers are assumed to be heterogeneous with respect to their capabilities. In general, it is assumed that the processing, networking, and imaging capabilities improve as proceed from a lower tier to a higher tier, at the expense of increased power consumption. Consequently, to maximize application lifetime, the overall application should use tier-specific resources judiciously and should execute its tasks on the most energy-efficient tier that has sufficient resource to meet the needs of that task. Thus, different tasks will execute on different tiers and various tiers of camera sensor network will need to interact and coordinate to achieve application goals. Given these intra- and inter-tier interactions, application design becomes more complex—the application designer needs to carefully map various tasks to different tiers and carefully design the various interactions between tasks.

One of the goals of SensEye is to illustrate these tradeoffs while demonstrating the overall benefits of the multi-tier approach. To do so, SensEye assumes three-tier architecture. The lowest tier in SensEye comprises mote nodes equipped with 900 MHz radios and low-fidelity Cyclops or CMUcam camera sensors. The second SensEye tier comprises stargate nodes equipped with web-cams. Each stargate is equipped with an embedded 400 MHz XScale processor that runs Linux and a web-cam that can capture higher fidelity images than Tier 1 cameras. Each Tier 2 node also consists of two radios-an 802.11 radio that is used by stargate nodes to communicate with each other, and a 900 MHz radio that is used to communicate with motes in Tier 1. The third tier of SensEye contains a sparse deployment of high-resolution pan-tilt-zoom cameras connected to embed PCs. The camera sensors at this tier are retargetable and can be utilized to fill small gaps in coverage provided by Tier 2 and to provide additional redundancy for tasks such as localization. Nodes in each tier and across tiers are assumed to communicate using their wireless radios in *ad-hoc* mode. No base-stations are assumed in this environment. The radio interface is assumed to be duty-cycled to meet application requirements of latency and lifetime constraint at each node. Consequently, the application tasks need to be designed carefully since radios on the nodes are not always-on. The above system model presents the key design principles of SensEye.

#### 4.1.8. STONE

As the field of sensor networks has seen developing very fast in recent years, this project [53] focuses on the energy efficient storage for the sensors. In this case, sensor platforms are equipped with a finite energy source. Thus significant research is focused on optimizing the energy consumption of node resources such as computation, communication and storage. Since new generation of sensor platforms has tracked technology trends in computation and communication components, the project deals with the storage subsystem that has undergone little change. All generations of the Mica motes provide limited storage of a less than a megabyte, at an energy cost equivalent to or greater than that of communication. The high energy cost of storage has raised questions about the rationale for using in-network storage-based data management techniques for sensor networks; though a lot of current research assumes the presence of such an energy efficient storage subsystem. There are few key questions that the project aims to deal, (i) to find out the most energy-efficient storage platform for sensor networks and to ascertain the implications of an ultra-low power storage subsystem on sensor network design and (ii) to design a storage frame-work that allows sensor network researchers/developers to use ultra-low power storage in an efficient and useful manner.

#### 4.1.9. SenQ

SenQ is a multi-layered embedded query system for efficient, real-time data extraction from heterogeneous sensors [54,55]. It consists of nesC and Java implementations, and a lightweight network protocol. It has the capability to co-reside with a graphical library on a MicaZ-based embedded user interface. It enables user-driven and peer-to-peer in-network query issue by wearable interfaces and other resource-constrained devices. Complex virtual sensors and user-created streams can be dynamically discovered and shared, and SenQ is extensible to new sensors and processing algorithms. Efficiency and performance of SenQ’s have been evaluated in the AlarmNet test bed. It has shown that on-demand buffering, query caching, efficient restart and other optimizations reduce network overhead and minimize data latency. A key design decision was to layer SenQ so that it could be used in part of all of AlarmNet and other applications. The lowest layers can operate entirely between two sensor devices, such as between a wearable user interface and a body-worn sensor. Upper layers include support for management functions on a gateway, and a restricted query language for users.

#### 4.1.10. WebDust

WebDust [56] deals with a generic and modular environment that develops applications for wireless sensor networks. It allows the application implementor to create a customized environment that will provide a wide range of services for wireless sensor networks. This section presents open architecture of WebDust, the most important design decisions, and discusses its distinct features and functionalities. WebDust stores the information extracted from the wireless sensor network in a database. It offers extendable statistics and provides a set of web-based user interfaces that allow the designer to present the information in different ways based on the needs of the application. This environment allows automatic registration of motes and the devices technical characteristics in heterogeneous, hierarchical sensor networks. The main strengths behind WebDust are: (i) the administrator can easily setup and control the network as motes register themselves automatically. (ii) multiple, heterogeneous, wireless sensor networks can be controlled as a single, unified, virtual sensor network. (iii) many users can simultaneously query, monitor, and visualize the execution of the wireless sensor network through a Web-based user interface.

In most of the project, emphasizes on the following aspects of the sensor network have been encountered in connection with the virtualization of sensor networks:

- Networking Technology: A few sensor network virtualization prototypes have been developed for specific sensor networking technologies with an aim to exploit unique characteristics of these sensor networks to enable virtualization.
- Layer of Virtualization: Considering the present wireless sensor network, researchers have naturally approached network virtualization in a hierarchical manner. For this reason different projects have attempted to virtualize different layers of sensor network protocol stack. It is found as the design of an abstract layer.
- Architectural domain: Initially most of the researchers have focused on the WSN architecture domains, which dictate the design choices made in the construction of architectures and services that can be offered on those platforms.
- Level of virtualization: To enable virtualization, it is a must to virtualize the tiny sensor nodes, links, and other resources in the sensor network. The level of virtualization refers to the granularity at which each virtual node can administer itself.

## 5. Opportunities Provided by VSN

Virtualization of sensor network has provided a lot of opportunities. It has provided a new research paradigm and a perfect business model. Actually, no matter how interesting the concept of the sensor network virtualization may be from the technical point of view, it will only become a reality in commercial environments if there are enough opportunities for network providers to deploy it. Some scenarios in which an infrastructure provider may benefit from implementing sensor network virtualization could be the following:

### 5.1. Sharing of Physical Network

The most important opportunity behind sensor network virtualization is the sharing of substrate physical infrastructure. Along with a cost-reduction strategy, federated sensor network operators are steadily exploring the deployment of common infrastructures to share capital investments. Our business model in Figure 1 depicts that the same physical sensor network is shared by two virtual sensor network. The application level users are getting different types of services from the virtual service provider. This is a nice opportunity that can be provided by the VSN.

### 5.2. Reducing Complexity and Cost of Sensor Overlay Proliferations

It is very difficult to maintain different sensor networks for individual purpose. It increases the complexity of any application. On the other hand, it is very expensive and difficult to deploy overlay network upon a particular physical sensor network. In both cases VSN can provide a viable alternative which is suitable in terms of complexity and cost of proliferations. Sensor network providers may introduce virtualization of sensor networks for a variety of reasons like organizational issues, regulatory challenges, security, scalability, and quality of experience. If an organization does not implement VSN technology within its own domain, it needs to build separate networks for different services to maintain quality-of-service requirements.

### 5.3. Reselling Infrastructure to Third Parties

This service can be conceived as an enhancement of the VSN portfolio. Nevertheless, sensor networks may be considered as key assets from a large scale federated operator’s perspective. There may be different pricing expectations from different stakeholders. The development of novel compensation mechanisms will be necessary to ensure that each role finds a place in the value chain. Through the VSN approach federated sensor network infrastructure provider may share or resell the physical infrastructure to the third party. It may open a new business opportunity in the sensor network research.

### 5.4. Diversification of Infrastructure

Sensor network operators may use virtualization technology to diversify their own infrastructure for private purposes. The provider can also render services to trusted or corporate third parties. The aim would be to optimize the delivery of multiple isolated services and virtual sensor network architectures over a common, cost effective infrastructure. In this way opportunity for diversification of WSN infrastructure may be achieved by VSN technology.

### 5.5. Managed Services

Large operators of the federated sensor networks are increasingly focusing their growth strategies towards services delivery, customer orientation and product marketing. In this context, a potential approach would be the externalization of infrastructure to better focus on the core service oriented business. A third party could in this context become an infrastructure provider, and could therefore benefit from sensor virtualization techniques to better capitalize its investments in new sensor network deployments. The same approach could be followed by governments or public entities aiming at deploying common sensor infrastructure, to promote the development of the digital society. However, publicly managed sensor networks have proven to be a difficult task, mainly for political reasons.

### 5.6. Brings Flexibility and Scalability

Sensor Network virtualization brings a new dimension of flexibility and scalability to the network infrastructure. By introducing the virtualization concept it is possible to ensure the flexibility and scalability issues in the sensor network. It can easily make it possible for multiple heterogeneous sensor networks to coexist, that resolve the scalability issues. It also makes the network flexible.

### 5.7. Simplified Architecture

VSN allows a simplified architecture that serves all of the applications and networks such as sensing sound, temperature, motion, viewing the object, monitoring the environment, *etc*., which previously required individual specific purpose sensor networks and unnecessary duplication. So VSN provide a simplified heterogeneous architecture of sensor network.

### 5.8. Decoupling Services

Decoupling means separation of code block VSN from that should depend on each other. Some code blocks are generic and should not know details of others. Since virtualization of sensor network set a virtual boundary among the infrastructure provider and the service provider, it makes possible the decoupling of different types of services. With services decoupled from sensor networks, new services and virtual sensor networks can be introduced without building overlays.

### 5.9. New Business Models

The virtualization in sensor network allows for new business models. Several virtual sensor network service providers can launch diversified services and applications instead of investing in the physical sensor network. This can provide an economy of scale business model for the end users as well as the service providers.

### 5.10. Increased Profitability

The virtualization in sensor networks opens the potential for increased profitability. Through the VSN concept the same sensor infrastructure can be shared by different virtual service providers, resulting in increased profitability. The degree of profit increases as the service level increases in terms of sensor network as a service and software as a service.

In a research environment, virtual sensor network infrastructure providers could be promoted to permit the validation of emerging federated WSN architectures. In the long term, this approach could be followed by network operators aiming at providing enhanced peering models with the goal to increase the value for global connectivity services.

## 6. Applications of VSNs

Actually the development of wireless sensor networks was originally motivated by military applications such as battlefield surveillance. However, wireless sensor networks are now used in many civilian applications, including environment and habitat monitoring, healthcare applications, smart homes, and traffic control. In the following subsections we consider the application of VSNs in battlefield monitoring, rock slides and animal crossing and smart houses [22,57–61]. We have briefly explained all the applications in the field VSN with possible case studies in some cases. We do not provide all the cases in all the applications since it would greatly increase the span of the survey.

### 6.1. Battlefield Monitoring

Nowadays the world is facing many conflicts among different nations. It is leading the peaceful world to an unstable state which is not suitable for mankind and all the creations that exist on Earth. It costs huge resources and takes the life of civilians and other innocent beings which detrimental for society as well as the environment. It is also a major reason for worldwide economic recession. We can consider of a scenario in a mountain area battlefield where different types of target groups such as civilians, enemies, soldiers, important infrastructure and animals coexist [58,59]. In such a situation, military operations are very sensitive which usually causes negative effects on the society. In this case, virtualization of sensor networks can serve the multiple purposes of sensing the environment, civilians, soldiers, animals and other sort of important features. Deployment of sensor nodes in a particular area and their effective utilization through virtualization can be a very cost effective approach in today’s battlefield. In the virtualization of sensor networks the same physical substrate sensor nodes can be used in different virtual networks for sensing temperature, sound, and humidity. It also helps detecting civilians, opponents and soldiers.

**Figure 4.**
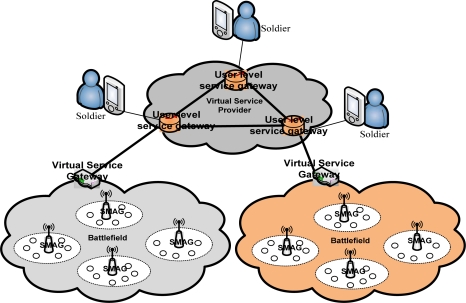
Application of VSNs in battlefield monitoring.

Sensor network virtualization in the battlefield can efficiently incorporate the overall scenario in a battlefield, as shown in the Figure 4. It can also be resource efficient, lessen the threat to civilians and can develop a new paradigm for efficient communication in the battlefield. Soldiers can monitor the battlefield by overseeing the scenario from virtual service providers for identifying sound, detection of the civilians and enemies, animals and the destructive weapons on the battlefield. This scheme can enhance the battlefield scenario as well as can decrease the cost of war and can save the innocent lives of civilian as well as the infrastructure. From a technical point of view, in a battlefield a lot of different types and multi-vendor sensors are deployed which include sound sensors, camera sensors, temperature sensors, motion detection sensors and other different types of sensors.

The battlefields are divided into different zones. In each zone, there are at least one or more gateway nodes. The gateway nodes work on behalf of the deployed sensor nodes. We can consider gateway based virtualization or individual sensor based virtualization. Gateway based virtualization is comparatively easier to implement. In case of individual sensor based virtualization techniques we should consider the resource constraints of the tiny sensor nodes. In either case a virtual service gateway provides virtual resources to the user level gateway.

The responsible troops can only access the user level service gateway and can perform operations as they desire. In gateway based virtualization, the gateway nodes receive different types of sensed data such as sound, temperature, motion and video. Thus, a gateway node holds different types of information and it can provide specific information to the particular virtual service gateway when necessary. In designing these types of battlefield scenarios, initially it could be expensive, but in the long run it will provide economies of scale and can be cost effective.

### 6.2. Rock Slides and Animal Crossing Monitoring

**Figure 5.**
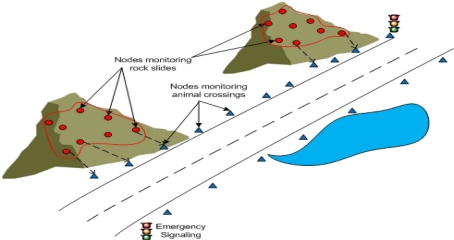
Applications of VSN in rock slide and animal monitoring.

VSNs can also be used in the application like rock sliding and animal crossing monitoring. Figure 5 represents a mountain terrain where there are different types of animals crossing a road.

In particular situations monitoring this type of event can be really critical. There may be rock slides on the mountain. To protect animals from rock slides, sensor nodes are deployed along the mountain areas [22]. There are emergency signaling systems to make the people and animals aware. A single physical WSN is deployed, but it is used by two VSNs. One VSN monitors rock slides and another VSN monitors animals crossing the mountainous terrain. Both applications rendered by the two VSNs use the same physical sensor nodes and relay the data to the signaling systems and/or to its members. In this scenario only two applications have been considered. Some other sophisticated applications may also be added to the existing system.

### 6.3. Smart House Monitoring

**Figure 6.**
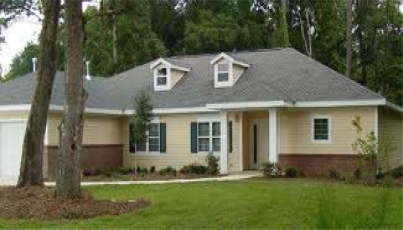
Gator Tech smart house (Courtesy: Gator Tech).

A smart house is a house that has highly advanced automatic systems for lighting, temperature control, multi-media, security, window and door operations, and many other functions. A smart home appears intelligent because its computer systems can monitor so many aspects of daily living. For example, the refrigerator may be able to inventory its contents, suggest menus, recommend healthy alternatives, and order groceries. The smart home systems might even take care of cleaning the cat's litter box and watering the plants. In Figure 6, we have demonstrated a smart house designed by the Gator Tech smart housing [60].

For the smart home automation and control system VSNs offer a wide range of services: local or remote access from the Internet to monitor the home [temperature, humidity, activation of remote video surveillance, status of the doors (locked or open) *etc.*] but also for home control (activate the air conditioning/heating, door locks, sprinkler systems, *etc.*). Fairly sophisticated systems can also optimize the level of energy consumption to a wide range of inputs from various sensors connected to the VSN: light sensors, presence detection sensors, temperature sensors, *etc.* in order to control electric window shades, chillers, air flow control, air that have direct interactions with the grid itself via the Internet of the grid network to report the amount of KWatts that could be load shed (home to grid) and to receive dynamic load shedding information if/when required (grid to home). This application is also referred to as Demand-Response application. Another service known as Demand Side Management could be provided by utilities to monitor and report to the user its energy consumption with a fine granularity (on a per device basis). Other inputs such as dynamic pricing can also be received by the user from the utility that can then turn on and off some appliances according to its local policy in order to reduce energy bills. In terms of home safety and security, the VSN can have motion- and audio-sensors, sensors at doors and windows, and video cameras to which additional sensors can be added for safety (gas, water, CO, Radon, smoke detection). The VSN typically comprises a few dozen of nodes forming an *ad-hoc* network with multi-hop routing since the nodes may not be in direct range. It is worth mentioning that the number of devices tends to grow considering the number of new applications for the home. In its most simple form, all nodes are static and communicate with a central control module but more sophisticated scenarios may also involve inter-device communication. For example, a motion/presence sensor may send a multicast message to a group of lights to be switched on, or a video camera will be activated sending a video stream to a gateway that can be received on a cell phone.

### 6.4. Structural Monitoring

Intelligent monitoring in facility management can make safety checks and periodic monitoring of the architecture status highly efficient. Powered nodes can be included in the design phase of a construction or battery-equipped nodes can be added afterwards. All nodes are static and manually deployed. Some data such as normal room temperature is not critical for security protection, but event-driven emergency data must be handled in very critical manner. Let us consider the following scenario for further clarification: a 1,000 m long bridge with 10 pillars is described. Each pillar and the bridge body contain five sensors to measure the water level, and five vibration sensors are used to monitor its structural health. The VSN nodes are deployed to have 100 m line-of-sight distance from each other. All nodes are placed statically and manually configured with a single-hop connection to the local coordinator. All VSN nodes do not move while the service is provided. The network configuration and forwarding/routing tables are changed only in case of node failure. Except from the pillars, there are no special obstacles to attenuate the node signals, but careful configuration is needed to prevent signal interference between VSN nodes. On the top part of each pillar, an “infrastructured” sink node is placed to collect the sensed data. The sink nodes of each pillar become data gathering points of the VSN hosts at the pillar as coordinators.

### 6.5. Healthcare

VSNs are envisioned to be heavily used in healthcare environments. Although hospital scenarios can be handled differently, VSNs provide great potential to ease the development of new services by getting rid of cumbersome wires and simplifying patient care in hospitals and for home care as the World is rapidly graying. The worldwide population of elderly people over age 65 is expected to be more than double from 357 million to 761 million by 2025 [6]. The speed with which this age-structural change is taking place implies an urgent need for solutions that will relieve the mounting pressure on our health-care systems as well as support a better quality of life and quality of care for our aged. Let us consider the following scenario for further clarification: an old citizen who lives alone wears one to a few wearable sensor nodes to measure heartbeat, pulse rate, *etc.* Dozens of sensor nodes are densely installed at home for movement detection. A WSN edge router at home will send the sensed information to a connected healthcare center. Portable base stations with LCDs may be used to check the data at home, as well. The different roles of devices have different duty-cycles, which affect node management. This scenario can be better handled by the concept of sensor network virtualization.

### 6.6. Vehicle Telematics

VSNs play an important role in intelligent transportation systems. Incorporated into roads, vehicles, and traffic signals, they contribute to the improvement of safety of transporting systems. Through traffic or air-quality monitoring, they increase the possibilities in terms of traffic flow optimization and help reducing road congestion. Let us consider the following scenario for further clarification: scattered sensor nodes are included in roads during their construction for motion monitoring. When a car passes over of these nodes, the possibility is then given to track the trajectory and velocity of cars for safety purposes. The lifetime of the sensor nodes incorporated into roads is expected to be as long as the lifetime of the roads. Multihop communication is possible between sensor nodes, and the network should be able to cope with the deterioration over time of the node density due to power failures. Sink nodes placed at the road side are mains-powered; sensor nodes in the roads run on battery. Power savings schemes might intermittently disconnect the nodes. A rough estimate of four nodes per square meter is needed. Other applications may involve car-to-car communication for increased road safety. This scenario can be better handled by the concept of sensor network virtualization.

### 6.7. Agricultural Monitoring

Accurate temporal and spatial monitoring can significantly increase agricultural productivity. Due to natural limitations, such as a farmers’ inability to check the crop at all times of day or inadequate measurement tools, luck often plays too large a role in the success of harvests. Using a VSN, indicators such as temperature, humidity, soil condition, can be automatically monitored without labor intensive field measurements. For example, VSNs could provide precise information about crops in real time, enabling businesses to reduce water, energy, and pesticide usage and enhancing environmental protection. The sensing data can be used to find optimal environments for the plants. In addition, the data on the planting condition can be saved by sensor tags, which can be used in supply chain management. Let us consider the following scenario for further clarification: in a fruit garden of medium to large size, a number of 50 to 100 sensor nodes are manually deployed in order to provide full signal coverage over the study area. An additional number of 100 to 1,000 leaf nodes with (possibly heterogeneous) specialized sensors (*i.e.*, humidity, temperature, soil condition, sunlight) are attached to the local wireless star topologies, periodically reporting measurements to the associated nodes. For example, in a 20-acre fruit garden with eight parcels of land, 10 sensor nodes are placed within each parcel to provide readings on temperature and soil moisture. The sensor nodes are able to support a multi-hop forwarding/routing scheme to enable data forwarding to a sink node at the edge of the fruits garden. Each of the eight parcels contains one data aggregator to collect the sensed data. Ten intermediate nodes are used to connect the sink nodes to the main gateway. This scenario can be better handled by the concept of sensor network virtualization.

### 6.8. Industrial Monitoring

VSN applications for industrial monitoring can be associated with a broad range of methods to increase productivity, energy efficiency, and safety of industrial operations in engineering facilities and manufacturing plants. Many companies currently use time-consuming and expensive manual monitoring to predict failures and to schedule maintenance or replacements in order to avoid costly manufacturing downtime. VSN can be inexpensively installed and provide more frequent and more reliable data. The deployment of VSNs can reduce equipment downtime and eliminate manual equipment monitoring that is costly to carry out. Additionally, data analysis functionality can be added to the network, eliminating the need for manual data transfer and analysis. Industrial monitoring can be largely split into the following application fields:

Process Monitoring and Control: combining advanced energy metering and sub-metering technologies with wireless sensor networking in order to optimize factory operations, reduce peak demand, ultimately lower costs for energy, avoid machine downtimes, and increase operation safety. A plant's monitoring boundary often does not cover the entire facility but only those areas considered critical to the process. Easy to install wireless connectivity extends this line to include peripheral areas and process measurements that were previously infeasible or impractical to reach with wired connections.

Machine Surveillance: ensuring product quality and efficient and safe equipment operation. Critical equipment parameters such as vibration, temperature, and electrical signature are analyzed for abnormalities that are suggestive of impending equipment failure.

Supply Chain Management and Asset Tracking: with the retail industry being legally responsible for the quality of sold goods, early detection of inadequate storage conditions with respect to temperature will reduce risk and cost to remove products from the sales channel. Examples include container shipping, product identification, cargo monitoring, distribution and logistics.

Storage Monitoring: sensor systems designed to prevent releases of regulated substances to ground water, surface water and soil. This application field may also include theft/tampering prevention systems for storage facilities or other infrastructure, such as pipelines.

From the above application points of view, it becomes apparent that VSNs are a most promising technology that can change our lifestyle. If we can provide a business model then this technology can be easily deployable to the customer.

## 7. Discussion

### 7.1. Available Solutions

Virtualization of sensor networks is a promising field of research. Recently the research community is paying attention to VSNs since it is cost effective in application development. Existing sensor network virtualization related research mostly focuses on fixing and enhancing some of the problems of the traditional wireless sensor network approaches. In this regard, VSNs deal with using sensor networks for supporting multiple applications, sensor overlay proliferation, developing tiny virtual machine for sensor networks, tiny sensor operating systems for VSNs, *etc.* Very recent different protocols are introduced by different projects that support VSNs. The Melete system is based on the Mate virtual machine that enables reliable storage and execution of concurrent applications on a single sensor node [16,25]. Recently the FRESnel project at Cambridge University aims to build a large scale federated sensor network framework with multiple applications sharing the same sensor node resources [59]. The primary aim of this project is to offer an environment that can support multiple applications running on each sensor node [27]. It provides an execution environment that hides from the running applications the fact that they operate in a shared environment. There are other different projects that also focus on VSN issues which have been discussed in Section 4.1 in some detail.

### 7.2. Future Research Scopes and Open Issues

Virtualization has further opened a new dimension in different research fields, especially in WSNs. The whole world is facing economic recession, so virtualization in sensor networks can be a promising research issue in the field of wireless sensor networks. Among the future research scopes few of them may be developing convenient operating systems for tiny sensors which can support virtualization in sensor networks. Managing resources, scheduling the sensing activities, minimizing energy consumption are a few of the future research areas in sensor network virtualization. Large scale federated sensor network frameworks with multiple applications sharing the same physical resources have already attracted the researchers’ attention. There are a lot of open research issues in the field of VSNs for example, problems related to connectivity between heterogeneous sensor nodes, service announcement and discovery, virtual sensor link embedding, virtual sensor node embedding, *etc.*

As a result, several technical challenges in terms of instantiation, operation, resource utilization and management of an overall sensor network virtualization environment remain unexplored, and many others require modification and improvement. Examples of instantiation related problems include interfacing, signaling, bootstrapping, and embedding of virtual sensor networks on shared physical sensor infrastructure; implementation of virtual sensor routers and virtual sensor links as well as resource scheduling among coexisting virtual sensor resources are a few of related future research issues. Finally, failure handling, mobility management, virtual sensor network configuration and monitoring are some examples of the management problems in the sensor network virtualization environment. In Section 3.2, we have discussed a wide range of open research challenges that need further exploration.

## 8. Conclusions

In this paper we have presented a survey on virtualization of wireless sensor networks. Virtualization in sensor networks could be effective in scenarios like smart home automation, patient monitoring, battlefield surveillance, rock slides and animal crossing in a mountainous terrain, among others. Multi-vendor sensor network architecture could be deployed for efficient utilization of physical sensor infrastructure. By allowing multiple heterogeneous wireless sensor network architectures to coexist on a shared physical substrate, virtualization of sensor networks might provide a new business model which could be cost effective in terms of deployment. Here we have summarized different project activities in the field of VSN research. We also introduced the design goal of VSN and discuss different challenges and opportunities of using the large scale federated WSN resources in a sensor virtualization environment. Our future interest is to build a large scale federated sensor network framework with multiple applications sharing the same resources. For this purpose we are working on the development of a virtual machine which is suitable for tiny sensor nodes and will facilitate true virtualization of wireless sensor networks.

## Figures and Tables

**Table 1.** Sensor network virtualization researches in different projects.

**Project**	**Research Area**	**URL**
FRESnel	To build a large scale federated sensor network framework with multiple applications sharing the same resources.	http://www.cl.cam.ac.uk/research/srg/netos/fresnel/index.html
VSNs	Random routing, virtual coordinates, and VSN support functions	http://www.cnrl.colostate.edu/Project/VSNs/vsns.html
SensorPlanet	SensorPlanet is a Nokia-initiated cooperation, a global research framework, on mobile device-centric large-scale Wireless Sensor Networks.	http://www.sensorplanet.org/
ViSE	Virtualization of sensor/actuator system, creating customized virtual sensor network test beds	http://groups.geni.net/geni/wiki/ViSE
STONE	Energy-efficient Storage for sensors	http://sensors.cs.umass.edu/projects/essense/
DVM	To build a system that supports software reconfiguration in embedded sensor networks at multiple levels	http://nesl.ee.ucla.edu/project/show/51
PRESTO	Takes a fresh look at the design of tiered large-scale sensor networks	http://presto.cs.umass.edu/
SensEye	Multi-tier multi-modal sensor networks	http://sensors.cs.umass.edu/projects/senseye/
SenQ	Complex virtual sensors and user-created streams can be dynamically discovered and shared.	http://www.cs.virginia.edu/wsn/medical/projects/senq
WebDust	Multiple, heterogeneous, wireless sensor networks can be controlled as a single, unified, virtual sensor network.	http://ru1.cti.gr/projects/webdust/wiki/JWebDust_application_enviroment
